# Expansion of Perceptual Body Maps Near – But Not Across – The Wrist

**DOI:** 10.3389/fnhum.2017.00111

**Published:** 2017-03-07

**Authors:** Matthew R. Longo

**Affiliations:** Department of Psychological Sciences, Birkbeck, University of LondonLondon, UK

**Keywords:** body representation, body image, tactile spatial remapping, touch, position sense, proprioception

## Abstract

Perceiving the external spatial location of touch requires that tactile information about the stimulus location on the skin be integrated with proprioceptive information about the location of the body in external space, a process called *tactile spatial remapping*. Recent results have suggested that this process relies on a distorted representation of the hand. Here, I investigated whether similar distortions are also found on the forearm and how they are affected by the presence of the wrist joint, which forms a categorical, segmental boundary between the hand and the arm. Participants used a baton to judge the perceived location of touches applied to their left hand or forearm. Similar distortions were apparent on both body parts, with overestimation of distances in the medio-lateral axis compared to the proximo-distal axis. There was no perceptual expansion of distances that crossed the wrist boundary. However, there was increased overestimation of distances near the wrist in the medio-lateral orientation. These results replicate recent findings of a distorted representation of the hand underlying tactile spatial remapping, and show that this effect is not idiosyncratic to the hand, but also affects the forearm. These distortions may be a general characteristic of the mental representation of the arms.

## Introduction

Several classes of afferent signals provide information about the current postural configuration of the body, including receptors in joints, in muscle spindles, and in the skin signaling skin stretch (Burgess et al., 1982; Proske and Gandevia, 2009, 2012). Efferent copies of motor commands also provide information used to determine current limb position (Gandevia et al., 2006; Walsh et al., 2013). There is thus clear physiological evidence that perceived limb position is influenced by receptors in joints (e.g., Ferrell et al., 1987; Macefield et al., 1990), in muscle spindles (e.g., Goodwin et al., 1972; Matthews, 1972), and in the skin (e.g., Edin and Johansson, 1995; Collins et al., 2005). There is, thus, a diverse set of afferent signals which shape the perception of limb position.

Critically, however, each of these signals provides information about the *angles* of joints, rather than their absolute location in space. Nevertheless, explicit judgments of body part location appear more precise than judgments of limb angles (Fuentes and Bastian, 2010). Similarly, neurons in somatosensory cortex (Prud’homme and Kalaska, 1994; Tillery et al., 1996), posterior parietal cortex (Kalaska et al., 1990), and motor cortex (Graziano et al., 2002) show selectivity for end-point positions, rather than individual joint angles. Thus, raw afferent information specifying joint angles is converted into a representation of absolute position in egocentric space. Calculating the absolute spatial location of part of the body requires that these signals specifying angular information be combined with information about the size and shape of the body segments between joints, information not specified by any afferent signal, or combination of signals. We therefore argued that position sense requires that immediate sensory signals be combined with a stored, central representation of the metric properties of the body, which we called the *body model* (Longo et al., 2010).

Longo and Haggard (2010) developed a procedure to isolate and measure this body model in the case of the hand. Participants placed their hand palm down on a table underneath an occluding board. They then used a long baton to judge the perceived location of the tip and knuckle of each finger. An overhead camera recorded the locations of judgments. By comparing the relative locations of judgments of each landmark, we constructed perceptual maps of hand structure underlying position sense. These maps were grossly distorted, featuring overall underestimation of finger length (i.e., the distance between the knuckle and tip), a radial-ulnar gradient with finger length underestimation increasing from the thumb to little finger, and overall overestimation of hand width (i.e., the distance between pairs of knuckles). Thus, position sense appears to rely on a highly distorted representation of body size and shape, with the hand represented as substantially wider than it actually is. Similar results have been found in several subsequent studies (Longo and Haggard, 2012a,b; Longo et al., 2012; Lopez et al., 2012; Ferrè et al., 2013; Longo, 2014; Saulton et al., 2014, 2016; Coelho et al., 2016).

In three recent studies (Mattioni and Longo, 2014; Longo et al., 2015b; Longo and Morcom, 2016), we have extended this hand-mapping paradigm to investigate the integration of proprioceptive and tactile information involved in localizing touch in external space, a process known as *tactile spatial remapping* (Yamamoto and Kitazawa, 2001; Azañón and Soto-Faraco, 2008; Bremner et al., 2008; Heed and Azañón, 2014; Heed et al., 2015). Mattioni and Longo (2014) compared perceptual maps of hand structure obtained by localizing the knuckles and fingertips either by verbal instruction, as in the studies described in the previous paragraph, or by a tactile stimulus being applied to that location. Broadly similar patterns of distortion were apparent in both conditions, though with some differences in magnitude. Because tactile stimuli in this study were always applied to linguistically labeled landmarks (i.e., the knuckles and fingertips), it is possible that rather than localizing the location of the touch in external space, participants merely took the touch as a cue for which landmark to localize. In a subsequent study, Longo et al. (2015b) used a similar paradigm but applied touch to several non-landmark locations on the hand dorsum, organized in a 3x3 grid. We compared the overestimation of distances between pairs of landmarks oriented in the proximo-distal hand axis with those oriented in the medio-lateral axis. There was modest overestimation of distances in the proximo-distal orientation (approximately 10–20% of actual distance), but substantially larger overestimation of distances in the medio-lateral orientation (approximately 40–80% of actual distance). Longo and Morcom (2016) replicated this result using a 4x4 grid of points. These results suggest that tactile spatial remapping relies on a distorted representation of the hand, wider and squatter than its actual shape, a pattern broadly similar to the pattern found for position sense alone, described in the previous paragraph.

The exact mechanisms underlying these distortions remain uncertain. Several lines of evidence, however, suggest that the distortions have a central origin. For example, Longo et al. (2012) found highly similar maps in a woman born without a left arm but with periodic phantom experiences, both for her intact right hand and for her ‘phantom’ left hand. Similarly, Ganea and Longo (2017) found highly similar maps when participants judged the location of landmarks on their actual hand under the board and when they merely imagined their hand being there. Further, some aspects of the distortions (e.g., the underestimation of finger length) appear when participants use visual memory to judge the remembered location of landmarks on a rubber hand (Longo et al., 2015c; Saulton et al., 2016), or even non-body objects (Saulton et al., 2014). Studies using a wide-range of methods have suggested that the posterior parietal cortex is involved in combining inputs from different modalities to construct estimates of limb position (e.g., Sakata et al., 1973; Graziano et al., 2000; Lloyd et al., 2003; Bolognini and Maravita, 2007; Azañón et al., 2010; Fautrelle et al., 2013), though some others implicate the premotor cortex (e.g., Graziano, 1999; Valenza et al., 2004). Integration of sensory signals with a stored body model may thus occur at these sites of multisensory integration in computing limb position.

Several aspects of these distortions appear to parallel aspects of the organization of the somatosensory cortices. For example, the progressive decrease in the represented length of the fingers from the thumb to little fingers (Longo and Haggard, 2010) mirrors gradients in both cortical magnification (Duncan and Boynton, 2007) and acuity (Vega-Bermudez and Johnson, 2001; Duncan and Boynton, 2007) of the different fingers. Similarly, the overestimation of distances in the medio-lateral axis relative to the proximo-distal axis is mirrored by the fact that tactile acuity is higher in the medio-lateral axis of the limbs (e.g., Weber, 1834/1996; Cody et al., 2008) and that the perceived distance between two touches is increased in the medio-lateral axis (e.g., Green, 1982; Longo and Haggard, 2011). Each of these effects of orientation may be related to the fact that the receptive fields (RFs) of neurons in primary somatosensory cortex representing the limbs tend to be oval-shaped, rather than circular, with the long axis of the oval running along the proximo-distal limb axis (e.g., Powell and Mountcastle, 1959; Brooks et al., 1961; Alloway et al., 1989). Given that the spacing between the RFs of adjacent neurons is a constant proportion of RF size (Sur et al., 1980), oval-shaped RFs should lead to denser spacing of RFs in the medio-lateral axis of limb. We recently suggested that individual RFs function as ‘pixels’ in maps of the body, with distance determined by essentially counting the number of RFs between two locations (Longo and Haggard, 2011; Longo, 2017). Because RFs are smaller across the medio-lateral axis of the limbs, distances oriented with this axis will have more unstimulated ‘pixels’ than distances along the proximo-distal limb axis, and thus may be considered as farther apart.

To date, studies using these paradigms have focused specifically on the hand. In this study, I extended this approach to the forearm. The motivation for this was twofold. First, I aimed to determine whether the distortions described above are idiosyncratic to the hand, or affect other body parts as well. Second, I aimed to see how the presence of the boundary between the two body parts, the wrist joint, affects the represented spatial layout of the body. Joints have been argued to be critical for providing spatial structure to the body, providing the “hinges” for segmenting the body into distinct parts (Bermúdez, 1998). Studies of tactile localization have found that joints function as reference points, with localization error being reduced near the shoulder, elbow, and wrist joints (Weber, 1834/1996; Boring, 1942; Cholewiak and Collins, 2003). Perhaps related to increased localization accuracy, other studies have shown heightened tactile spatial acuity in the immediate vicinity of the wrist than on the adjacent skin of the forearm and hand (Cody et al., 2008). Most pertinent in the present context, two recent studies have argued for categorical perception effects caused by the wrist boundary (de Vignemont et al., 2009; Le Cornu Knight et al., 2014). de Vignemont et al. (2009) obtained verbal estimates of perceived tactile distance, finding that these were increased when the two touched locations fell on opposite sides of the wrist (i.e., one on the hand and one on the forearm) than when both stimuli were applied entirely on either body part. Le Cornu Knight et al. (2014) asked participants to make forced-choice judgments of which of two tactile distances felt larger, one oriented in the proximo-distal limb axis and the other in the medio-lateral axis. They found that the baseline bias for medio-lateral distances to be perceived as larger than proximo-distal ones (Green, 1982; Longo and Haggard, 2011; Canzoneri et al., 2013; Miller et al., 2014) was reduced at the wrist, consistent with perceptual expansion of distances that crossed the wrist boundary.

The procedures were similar to those used by Longo et al. (2015b), but used an expanded set of stimulation locations. In the *Tactile Task*, a 4x2 grid of locations was made in ink on the participant’s left arm using a stencil, resulting in one 2x2 grid entirely on the hand dorsum and another 2x2 grid entirely on the forearm. This allowed comparisons of overestimation of distances entirely on the hand, entirely on the dorsum, and crossing the joint boundary between the two body parts. For comparison, I also measured perceptual hand maps by verbally instructing participants to localize the knuckles and fingertips of each finger (*Verbal Task*).

## Materials and Methods

### Participants

Twelve individuals (eight females; mean age: 21.8 years; *SD*: 2.8 years) participated after giving informed consent. All participants were right-handed as assessed by the Edinburgh Inventory (Oldfield, 1971) (*M*: 77.97; range: 33.33–100). The study was conducted in accordance with the principles of the Declaration of Helsinki with written informed consent from all participants. Procedures were approved by the Department of Psychological Sciences Research Ethics Committee at Birkbeck, University of London.

### Procedure

Procedures were similar to those used in previous studies with this paradigm (Longo and Haggard, 2010, 2012a,b). Participants sat with their left hand and forearm resting palm-down on a table. An occluding board (40 cm × 40 cm) was placed on four pillars (6 cm high) to occlude the hand. A webcam (Creative Live Cam Voice) was suspended from a tripod 27 cm above the table and captured photographs (1280 × 960 pixels) under control of a custom Matlab (Mathworks, Natick, MA, USA) script. Fisheye distortion in the photographs was corrected using the Panotools plug-in^1^ for Adobe Photoshop CS2.

In the *Verbal Task*, the participant used a long baton (35 cm length; 2 mm diameter) to indicate the perceived location of landmarks on their left hand, underneath the occluding board. Ten landmarks were used: the tip of each finger (i.e., the most distal point) and the knuckle of each finger (i.e., the center of the knuckle at the base of each finger). On each trial, participants were instructed verbally which landmark to localize. They were asked to be precise in their responses, to avoid ballistic movements, and to refrain from strategies such as tracing the outline of the hand. To ensure that responses were independent, participants moved the tip of the baton to the blue dot at the edge of the occluding board after each trial. When participants indicated that they were happy with their response, a photograph was captured showing their response and stored for offline coding. At the beginning and end of each block a photograph was taken without the occluding board to allow measurement of actual hand structure and to ensure that the hand and arm had not moved during the block. A 10 cm ruler appeared on the table in the images without the occluding board, allowing conversion between distances in pixels and cm. To facilitate coding of the actual location of the knuckles, a small black mark was made with a pen on each knuckle at the start of the experiment by asking the participant to make a fist.

The *Tactile Task* was similar to our recent studies (Longo et al., 2015b; Longo and Morcom, 2016), the participant was touched with a wooden stick at one of eight locations on their left hand or forearm and judged the perceived location of touch. The locations were arranged in a 4x2 grid, centered on the wrist, so that there was one 2x2 grid on the hand dorsum and another on the forearm. The distance between adjacent points was 3.5 cm. The locations were marked with a black pen using a flexible plastic stencil. The tactile stimulus was applied manually by an experimenter, who lifted up the occluding board to access the hand/arm, tilting the board toward the participant to prevent their seeing the location of the stimulus. The tactile stimulus was applied for approximately 1 s.

There were two experimental blocks of each task, in ABBA order, with the initial condition counterbalanced across participants. Within each block, there were three repetitions of each landmark (in the verbal task) or stimulus location (in the tactile task). The trials within a block were arranged in three sequential mini-blocks, each including one trial of each location, in random order. There were thus 30 trials per block in the verbal condition and 24 trials per block in the tactile condition.

### Analysis

Analysis methods were similar to our previous studies using this paradigm. The x-y pixel coordinates of the actual location of each landmark (in the verbal task) and stimulus location (in the tactile task) were coded offline from the images without the occluding board. In addition, the pixel coordinates of each end of the ruler were coded, allowing us to convert distances from pixels to cm. Similarly, the judged location of each landmark or stimulus location was coded by the x-y pixel location of the tip of the baton.

For our statistical analysis, we calculated the distance between pairs of locations or judgments using the Pythagorean theorem and converted these to cm. We then calculated overestimation as a percentage of actual size using the following formula: Percentage Overestimation = 100 ^∗^ (Judged Distance – Actual Distance)/Actual Distance.

**FIGURE 1:**
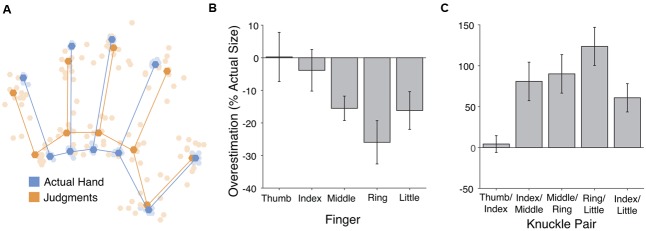
**Results from the verbal task. (A)** Perceptual hand maps from individual participants (pale orange dots) in the verbal task placed in mutual Procrustes alignment with each other and with maps of actual hand shape (pale blue dots). The dark dots and lines show the grand average shape of perceptual maps (dark orange) and actual hand structure (dark blue). **(B)** Overestimation of finger length. Across fingers there was overall underestimation of finger length, which increased from the radial (thumb) side of the hand to the ulnar (little finger) side. **(C)** Overestimation of distance between pairs of knuckles. Hand width was substantially overestimated. Error bars are one standard error of the mean.

**FIGURE 2:**
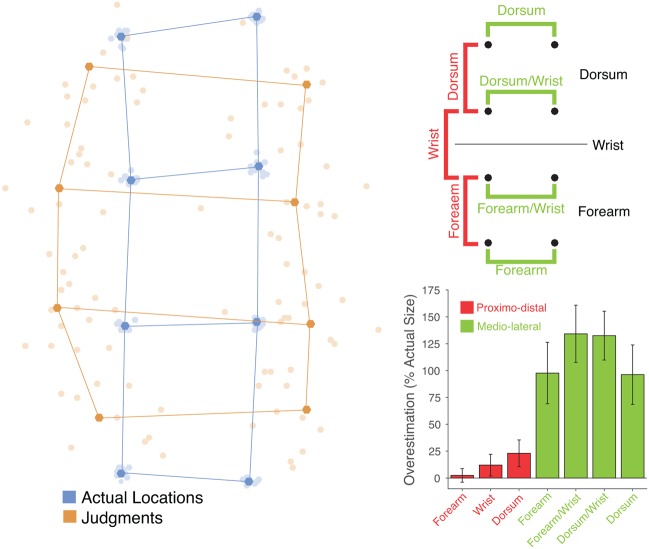
**(Left)** Perceptual maps from individual participants (pale orange dots) in the tactile task placed in mutual Procrustes alignment with each other and with maps of actual hand shape (pale blue dots). The dark dots and lines show the grand average shape of perceptual maps (dark orange) and actual hand structure (dark blue). **(Top right)** Schematic of the eight points showing the distances that were calculated. In the proximo-distal orientation (red) distances were calculated between locations entirely on the dorsum, entirely on the forearm, or crossing the wrist. In each case, distances between pairs of locations in the left and right columns were averaged. In the medio-lateral orientation distances were calculated between locations fully on the dorsum or forearm (top and bottom rows) and locations near the wrist (middle two rows). **(Bottom right)** Overestimation of distance as a percentage of actual distance for each type of distance. Distances in the proximo-distal orientation are shown in red, and those in the medio-lateral orientation in green. There was substantial overestimation of medio-lateral distances, but only modest overestimation of proximo-distal distances.

To graphically display perceptual maps, we used Procrustes alignment (Rholf and Slice, 1990; Bookstein, 1991), which scales, translates, and rotates configurations of homologous landmarks to superimpose them as much as is possible. Because the fingers are articulated and can move independently, differences in hand shape can be confounded by differences in hand posture (Adams, 1999). We therefore rotated the fingers of each hand to a common posture, defined for each finger as the angle formed by the intersection of the line running through the knuckles of the index and little fingers and the line running between the tip and knuckle of a particular finger. These angles were 39.6, 64.4, 76.5, 87.1, and 108.8°, for the thumb through little fingers, respectively. Because there were two experimental blocks of each condition, I first put the two maps of each type for a given participant into mutual Procrustes alignment and took the grand-average shape. These maps were then placed-into a second-level Procrustes alignment across participants, as shown in **Figures 1, 2**.

## Results

### Verbal Task

The left panel of **Figure 1** shows the perceptual maps, placed into Procrustes alignment to allow calculation of a grand-average shape. In our previous studies using this paradigm we have found three specific patterns of distortion of these perceptual maps: (1) overall underestimation of finger length, (2) a radial-ulnar gradient with underestimation increasing from the thumb-side to the little finger-side of the hand, and (3) overestimation of hand width. All of these were replicated in the present study. First, collapsing across the five fingers, there was significant underestimation of finger length (*M*: -12.25%), *t*(11) = -2.85, *p* < 0.02, *d* = 0.824 (see **Figure 2**, center panel). An analysis of variance (ANOVA) revealed that the magnitude of underestimation differed across the five fingers, *F*(4,44) = 4.55, *p* < 0.005, $\eta_{P}^{2}$ = 0.293. We quantified the change across the five fingers using least-squares regression, regressing percent overestimation on finger number (i.e., thumb = 1 to little finger = 5). There was a significant gradient, with underestimation increasing from the thumb toward the little finger (*M*: -5.50% per finger), *t*(11) = -2.82, *p* < 0.02, *d* = 0.815. Taking the distance between the knuckles of the index and little fingers as an overall measure of hand width, there was clear overestimation of hand width (*M*: 60.8%), *t*(11) = 3.52, *p* < 0.005, *d* = 1.015 (see **Figure 2**, right panel).

Finally, as an overall measure of the aspect ratio of the hand, we calculated the *shape index*, which we adapted from Napier (1980). The shape index is defined as 100 ^∗^ hand width/hand length. A large shape index thus indicates a wide, squat hand, and a small index a long, slender hand. As a measure of hand width we used the distance between the knuckles of the index and little fingers. As a measure of hand length we used the length of the middle finger. Shape indices were calculated both for the actual shape of participants’ hands and for the shape of perceptual maps. Shape indices were clearly larger in perceptual maps (*M*: 119.81) than actual hands (*M*: 61.44), *t*(11) = 4.25, *p* < 0.002, *d* = 1.23, again showing a clear bias for the hand to be represented as broader than its actual shape.

### Tactile Task

**FIGURE 3:**
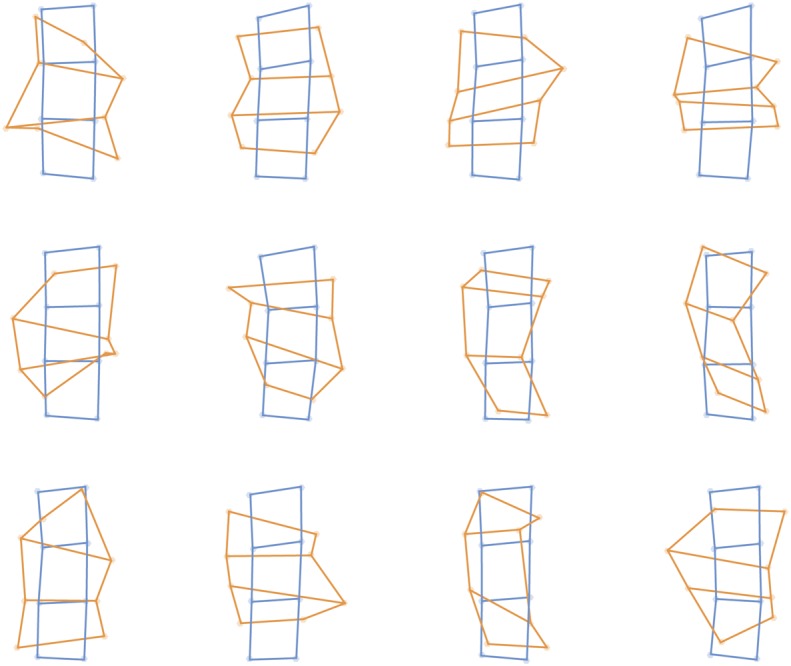
**Perceptual maps from individual participants in the tactile task (orange) placed into Procrustes alignment with maps of actual stimulus locations (blue), separately for each of the 12 participants**.

The left panel of **Figure 2** shows perceptual maps in the tactile task placed into mutual Procrustes alignment across participants and for both judgments and actual stimulus locations. **Figure 3** shows the same data separately for each participant. I calculated the distance between adjacent locations in either the proximo-distal or medio-lateral orientation. Overall, there was modest overestimation of distances in the proximo-distal orientation (*M*: 12.47%), *t*(11) = 2.16, *p* = 0.0537, *d* = 0.624 (the red bars in the bottom left panel of **Figure 2**), and substantial overestimation in the medio-lateral orientation (*M*: 115.11%), *t*(11) = 4.63, *p* < 0.001, *d* = 1.34 (the green bars in the bottom left panel of **Figure 2**). The magnitude of overestimation was significantly larger in the medio-lateral than in the proximo-distal orientation, both overall, *t*(11) = 4.72, *p* < 0.001, *d_z_* = 1.361, and considering stimuli on the dorsum (114.34 vs. 23.00%), *t*(11) = 5.68, *p* < 0.0001, *d_z_* = 1.641, and on the forearm (115.88 vs. 2.42%), *t*(11) = 4.51, *p* < 0.001, *d_z_* = 1.303, separately. An ANOVA on percent overestimation including orientation (medio-lateral vs. proximo-distal) and body part (dorsum vs. forearm) revealed a clear main effect of orientation, *F*(1,11) = 29.53, *p* < 0.001, $\eta_{P}^{2}$ = 0.729, but no effect of body part, *F*(1,11) = 2.21, *n.s.*, $\eta_{P}^{2}$ = 0.168, nor an interaction, *F*(1,11) = 1.36, *n.s.*, $\eta_{P}^{2}$ = 0.110. Thus, these results show that that the distortions found for perceptual maps underlying tactile spatial remapping reported by Longo et al. (2015b) are not idiosyncratic to the hand, but also affect the forearm. Moreover the distortions are of similar magnitude on the two body parts.

I next investigated the effects of the wrist on these distortions. As shown in the top left panel of **Figure 2**, I divided distances in the proximo-distal orientation into three categories, those entirely on the dorsum, those entirely on the forearm, and those crossing the wrist, shown in the red bars in the bottom left panel of **Figure 2**. An ANOVA on these data revealed no significant effect of location, *F*(1.26,13.84) = 1.08, *n.s.*, $\eta_{P}^{2}$ = 0.090. As can be seen in the figure, there was no hint of a perceptual expansion across the wrist boundary.

I similarly divided distances in the medio-lateral orientation into four categories, each associated with one row of the 4x2 grid, as seen in the top left panel of **Figure 2**. An ANOVA revealed a significant main effect of location, *F*(3,33) = 4.04, *p* < 0.02, $\eta_{P}^{2}$ = 0.269. The two locations adjacent to the wrist showed more overestimation than the locations farther from the wrist. There were significant increases for the location near the wrist both on the dorsum (132.51 vs. 96.16%), *t*(11) = 2.23, *p* < 0.05, *d_z_* = 0.643, and on the forearm (134.15 vs. 97.61%), *t*(11) = 2.37, *p* < 0.05, *d_z_* = 0.684. Thus, in contrast to the lack of expansion across the wrist boundary, there was a clear increase of overestimation for stimuli near the wrist oriented parallel to the wrist.

## Discussion

These results replicate the finding of Longo et al. (2015b) that tactile spatial remapping of stimuli on the hand relies on a distorted representation of body size and shape, with substantial overestimation of hand width compared to length. Further, they show that similar distortions appear on the arm, showing that this effect is not idiosyncratic to the hand. Thus, the localization of tactile stimuli applied to both the hand and the forearm relies on a similarly distorted representation of body size and shape. Interestingly, there was no expansion of distance across the wrist boundary, as might have been expected given recent studies reporting categorical perception of tactile distance across this joint (de Vignemont et al., 2009; Le Cornu Knight et al., 2014). There was evidence, however, for perceptual expansion for stimuli near the wrist oriented in the medio-lateral orientation (i.e., parallel to the wrist).

The broadly similar distortions found on the hand and forearm suggest that both may be integrated into a more general representation of the arm as a whole. Studies of perceived tactile distance have found qualitatively similar anisotropies on both body parts (Green, 1982; Longo and Haggard, 2011; Miller et al., 2014), with pairs of tactile stimuli oriented with the medio-lateral limb axis being perceived as farther apart than pairs oriented with the proximo-distal axis. Thus, for both tactile spatial remapping and tactile distance perception there appears to be a general bias to perceived the limb as wider than it actually is, affecting both the forearm and hand, consistent with the idea that both rely on a common body model (Longo et al., 2010). Although, one recent study found no correlation across participants in the magnitude of biases in the two modalities (Longo and Morcom, 2016).

In the case of tactile distance perception, however, there is also evidence that this perceptual anisotropy is even bigger on the forearm than on the hand (Le Cornu Knight et al., 2014; Miller et al., 2016). The current study did not find any evidence for differences between these body parts, with quite similar distortions apparent in both cases. Another important difference between the current study and previous studies of tactile distance perception was the absence of perceptual expansion across the wrist boundary, which has been reported in two recent studies of tactile distance perception (de Vignemont et al., 2009; Le Cornu Knight et al., 2014). The exact meaning of these differences between tactile spatial remapping and tactile distance perception is not fully clear. One possibility is that the two abilities may rely on body representations which, though similarly distorted, are nevertheless distinct. There is some existing evidence that this may be the case. For example, in both position sense (Longo and Haggard, 2012a) and tactile distance perception (Longo and Haggard, 2011; Longo et al., 2015a), distortions are smaller on the glabrous skin of the palm than on the hairy skin of the hand dorsum. But while the magnitude of distortion is strongly correlated on the two sides of the hand for position sense (Longo and Haggard, 2012a), they are uncorrelated for tactile distance perception (Longo et al., 2015a), which could indicate that tactile distance perception relies on a more fragmented representation of individual skin surfaces, while position sense relies on a more holistic, integrated representation of entire body parts (Longo, 2015a). If position sense relies on a more holistic representation of the entire arm than does tactile distance perception, this could account for why the wrist did not produce the same categorical perception effect in the current study as in previous studies of tactile distance perception (de Vignemont et al., 2009; Le Cornu Knight et al., 2014).

Another possible interpretation of the difference between the present results and those measuring tactile distance perception is that participants in this study made completely independent judgments of each stimulus location, whereas tactile distance judgments intrinsically involve a comparison of two distinct locations. That is, in the current study participants made a judgment on each trial about the *absolute* location in space of a single tactile stimulus. In contrast, in the studies of de Vignemont et al. (2009) and Le Cornu Knight et al. (2014), participants made judgments about the *relative* location of simultaneously presented tactile stimuli. It is possible that categorical perception effects will show up more strongly for comparative judgments of multiple stimuli (as in tactile distance judgments) than for absolute judgments of single stimuli (as in the current study).

Another possible explanation for the absence of an effect of crossing the wrist in the present study could be that participants simply don’t recognize exactly where the wrist actually is. We have recently reported large misunderstandings about the location of other joints in the hand – the knuckles – which people appear to believe are substantially farther forward in the hand than they actually are (Longo, 2015b; Margolis and Longo, 2015). Indeed, we found that the magnitude of this bias was correlated across participants with the extent of underestimation of finger length in the hand mapping task (Longo et al., 2015c). In the current study, were participants to misperceive the location of the wrist, it might be relatively unsurprising that no categorical perception effect was found. There are several reasons, however, to consider this interpretation unlikely. First, this issue would seem to pose equal complications for finding categorical perception effects in tactile distance perception, which have nevertheless been found in recent studies (de Vignemont et al., 2009; Le Cornu Knight et al., 2014). Second, in the current study the wrist did appear to affect performance, specifically leading to increased overestimation in the medio-lateral hand axis for stimuli presented near the wrist, suggesting that the location of the wrist had not been misperceived. Third, it seems likely that the misperception of knuckle location is at least partly related to the fact that the location of the knuckles is visually apparent only on the dorsal hand surface, but not on the palm. The wrist is very different in this sense, since it is visually apparent on both sides of the arm in terms of creases in the skin and an overall change in the shape of the limb.

## Conclusion

The present results show that similar distortions of body structure characterize tactile spatial remapping on the hand and forearm. This suggests that rather than being an idiosyncrasy of the hand, these distortions may reflect the general organization of the mental representation of the limbs. Indeed, in the case of tactile distance perception, similar biases appear on the legs as on the arms (Green, 1982). Moreover, there are cases in which the finger agnosia seen in the Gerstmann syndrome extends to the toes (Tucha et al., 1997), suggesting a common representation of digits across all limbs. It would be interesting in future research to directly compare the upper and lower limbs, given their clear serial homology, yet vastly different functional roles in everyday behavior.

## Author Contributions

ML designed the study, collected and analyzed the data, and wrote the paper.

## Conflict of Interest Statement

The author declares that the research was conducted in the absence of any commercial or financial relationships that could be construed as a potential conflict of interest.

## Footnotes

1 http://www.panotools.org/
